# Context-driven activation of odor representations in the absence of olfactory stimuli in the olfactory bulb and piriform cortex

**DOI:** 10.3389/fnbeh.2014.00138

**Published:** 2014-04-29

**Authors:** Nathalie Mandairon, Florence Kermen, Caroline Charpentier, Joelle Sacquet, Christiane Linster, Anne Didier

**Affiliations:** ^1^Centre de Recherche en Neurosciences de Lyon, UMR CNRS 5292 INSERM 1028, Université Lyon1Lyon, France; ^2^Computational Physiology Lab, Neurobiology and Behavior, Cornell UniversityIthaca, NY, USA

**Keywords:** olfactory bulb, Zif268, cell mapping, conditioning, visual context, modeling

## Abstract

Sensory neural activity is highly context dependent and shaped by experience and expectation. In the olfactory bulb (OB), the first cerebral relay of olfactory processing, responses to odorants are shaped by previous experiences including contextual information thanks to strong feedback connections. In the present experiment, mice were conditioned to associate an odorant with a visual context and were then exposed to the visual context alone. We found that the visual context alone elicited exploration of the odor port similar to that elicited by the stimulus when it was initially presented. In the OB, the visual context alone elicited a neural activation pattern, assessed by mapping the expression of the immediate early gene zif268 (egr-1) that was highly similar to that evoked by the conditioned odorant, but not other odorants. This OB activation was processed by olfactory network as it was transmitted to the piriform cortex. Interestingly, a novel context abolished neural and behavioral responses. In addition, the neural representation in response to the context was dependent on top-down inputs, suggesting that context-dependent representation is initiated in cortex. Modeling of the experimental data suggests that odor representations are stored in cortical networks, reactivated by the context and activate bulbar representations. Activation of the OB and the associated behavioral response in the absence of physical stimulus showed that mice are capable of internal representations of sensory stimuli. The similarity of activation patterns induced by imaged and the corresponding physical stimulus, triggered only by the relevant context provides evidence for an odor-specific internal representation.

## Introduction

Sensory neural activity is highly context dependent and shaped by experience and expectation. For example, throughout the olfactory system, neural responses to odors are shaped by behavioral relevance of the odor (Kay and Laurent, 1999; Martin et al., 2004; Doucette and Restrepo, 2008; Doucette et al., 2011; Wilson and Sullivan, 2011), by previous experience (Buonviso and Chaput, 2000; Moreno et al., 2009; Wilson, 2009; Chaudhury et al., 2010) and task difficulty (Mandairon et al., 2006; Li et al., 2008). Changes in neural responses to odors can be seen as early as in the olfactory bulb (OB), the target of sensory neurons (Freeman and Schneider, 1982; Mandairon and Linster, 2009). Contextual information is presumably shaped by previous experience and expectation and mediated to first order sensory structures by feedback projections from higher brain areas. The OB is an ideal target structure for the integration of sensory and contextual information because it receives direct inputs from sensory neurons, without thalamic detour, as well as a massive inputs from higher order brain areas such as noradrenergic and cholinergic nuclei, amygdala, piriform cortex, enthorinal cortex (Shipley and Ennis, 1996). Inspired by paradigms used in human imagery experiments which showed that a visual stimulus previously associated with an odorant is able to activate primary olfactory cortical regions (Gottfried et al., 2002, 2004) in the absence of olfactory stimulation, we here tested if association of an odorant with a visual context in mice would allow the visual context alone to elicit a behavioral responses usually associated with an olfactory stimulus as well as neural activation in olfactory pathways. In the OB, odor quality is represented by distributed patterns of activity in both the glomerular and granule cell layers. Each odor is represented by a unique pattern of relative activity across the OB, as visualized by 2DG (Johnson and Leon, 2007) or immediate early gene (IEG) mapping (Inaki et al., 2002) as well as partial visualization using optical methods. In addition to these spatial activity patterns, odors also evoke unique temporal patterns across the OB, as well as stimulus induced oscillations and synchronization (Kay et al., 2009). Because activity in the granule cell layer is susceptible to activity dependent plasticity, is highly odor and experience specific, we here chose relative activation patterns of granule cells as a measure for odor representations in the OB. We find that in mice which had been presented with an odorant repeatedly in the same visual context, the visual context alone elicited a behavioral response similar to that elicited by the stimulus when it was initially presented. In the OB, the visual context alone elicited a neural activation pattern, assessed by mapping the expression of the immediate early gene zif268 (egr-1) that was highly correlated with that elicited by the associated odorant, but not other odorants. Both behavioral and neural activation was not elicited by a novel context and both dependent on intact feedback to the OB from higher brain areas. A computational model of the OB and cortex which incorporated known features of the interactions between these two areas showed that experimentally described plasticity in projections from cortex to bulb, paired with “context neurons” previously used in models of hippocampal processing (Hasselmo and Wyble, 1997) sufficed to reproduce the observed experimental results. We conclude that in rodents, neural representation of an odorant in primary sensory areas can be elicited in its absence by exposure to the context to which the odorant was previously associated. This further suggests that rodents can build internal representation of the olfactory stimulus.

## Methods

### Subjects

Sixty adult male C57BL/6J mice (8 weeks old, Charles River, L'Arbresles, France) were used in accordance with the European Community Council Directive of November 24, 1986 (86/609/EEC). Mice were kept in standard mouse cages with full access to food and water. Experimental group contained 5 to 13 animals.

### Experimental set up

**Figure 1:**
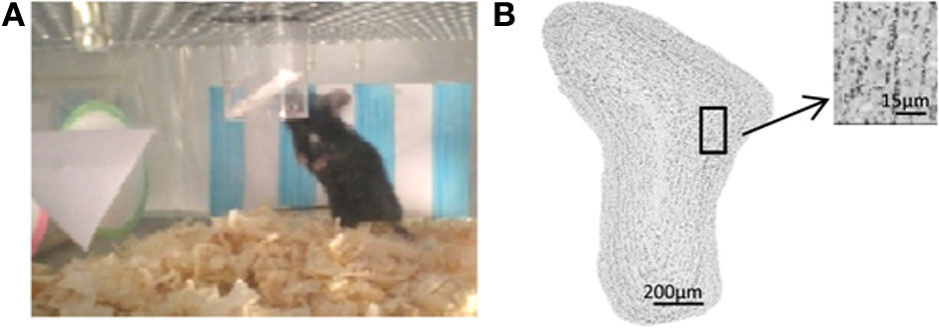
**Methodological aspects. (A)** Experimental cage. Visual cues are placed on the wall of the cage and a drilled plastic tube protruding from ceiling of the cage receives the odorized swab. (**B)** Zif268-positive cells in the granule cell layer of the OB.

All behavioral experiments were conducted in individual training cages (20 × 27 cm) with visual cues differing in shape, color and pattern added to the outside walls of the cage. The cage lids were pierced in their center and a 3-cm diameter tube was pushed inside the cage through this hole. The tubes were transparent and had 3-mm holes in their bottom through which odor diffused. This system allowed the introduction of the odor without opening the cages during the experiment (Figure 1A).

### Behavioral training

Thirty minutes per day during 10 days, mice were placed in the training cage (Figure 1A). Five minutes after their introduction, a non-odorized swab or an odorized swab was introduced into the tube.

For odorant presentation, the swab was impregnated with 60 μL of pure +Limonene (Sigma-Aldrich, purity 97%) or pure Decanal (Fluka, purity ≥95%).

### Behavioral test

On day 11, mice were placed again into the training cage or novel context cage. After 5 min, the non-odorized or odorized swab introduced, depending on the group. The amount of time that the mice investigated the tube was manually recorded during 10 min after the introduction of the stimulus (Figure 1A). Investigation was defined as active exploration within 1 cm around the odor port.

### Data analysis

All mice were included in the analyses. Results are expressed as mean ± s.e.m. For behavioral data, Kruskall–Wallis (multiple comparisons) Mann–Whitney (two-group comparisons) tests were applied. For Zif268 expression maps, data showed normal distribution and between-group differences were assessed using ANOVA followed by Fisher *post-hoc* tests for pair comparisons or unilateral *t*-tests when appropriate (Systat software). The level of significance was set to 0.05.

### Zif268 expression mapping

One hour after the test session, animals were killed by intracardiac perfusion (under deep anesthesia, Pentobarbital 3.64 mg/kg) and brains were sectioned using a cryostat. Zif268 immunochemistry (Mandairon et al., 2008) was performed for each animal on serial coronal sections (sampling interval = 70 μm). Zif268-positive cells were counted automatically in the granule cell layer of the OB using mapping software (Mercator, Explora Nova, La Rochelle, France; Figure 1B) coupled to a Zeiss microscope. The cell counts were conducted by experimenters who were blind to the experimental condition of the mice. The number of labeled profiles was divided by the surface of the region of interest to yield the total densities of labeled cells. Maps of Zif268-positive cells were constructed as previously described (Mandairon et al., 2006). Briefly, the granule cell layer was divided into 36 sectors of 10°. The number of labeled cells/μm^2^ was calculated for each sector and measurements were then merged into arrays of 10° × 70−μm bins yielding a 2-D map of the granule cell layer. Arrays were averaged within each group, and a colored image plot of the data was constructed. To compare odor and context evoked activation maps, we calculated the pairwise overlap between maps (Python scripts associated to the Scipy library). Following standard procedures, maps were first threshold to keep the 30% highest values (percentile). This threshold was set as best fitted to the clusters delineated by visual inspection of the Zif268 expression maps. We analyzed the similarities between maps by counting the number of overlapping pixels and calculating a percentage of overlap. Between groups comparisons were done using *t*-tests for comparison of proportions (Mandairon et al., 2006; Sultan et al., 2011).

Zif268 labeling was analyzed in the anterior piriform cortex (layer II) in about eight sections per mice (distributed between 1.18 and 2.46 mm anterior to Bregma, Paxinos Atlas). The boundary between the anterior and posterior cortex was located at the level of the anterior commissure.

### Cannulation

Mice were anesthetized (100 mg/kg ketamine and 6 mg/kg xylazine, i.p.) and implanted as described earlier (Kermen et al., 2011) into both olfactory peduncles at the following coordinates with respect to bregma: AP = +2.4 mm; ML = ± 0.75 mm; DV = −3 mm. Following surgery, mice were allowed to recover for 10 days before beginning the training. Lidocaine (Sigma) (2%, 1 μl/side) was freshly prepared and infused into the each medial peduncle. Behavioral testing began 10 min after lidocaine administration was completed.

### Computational modeling

**Figure 2:**
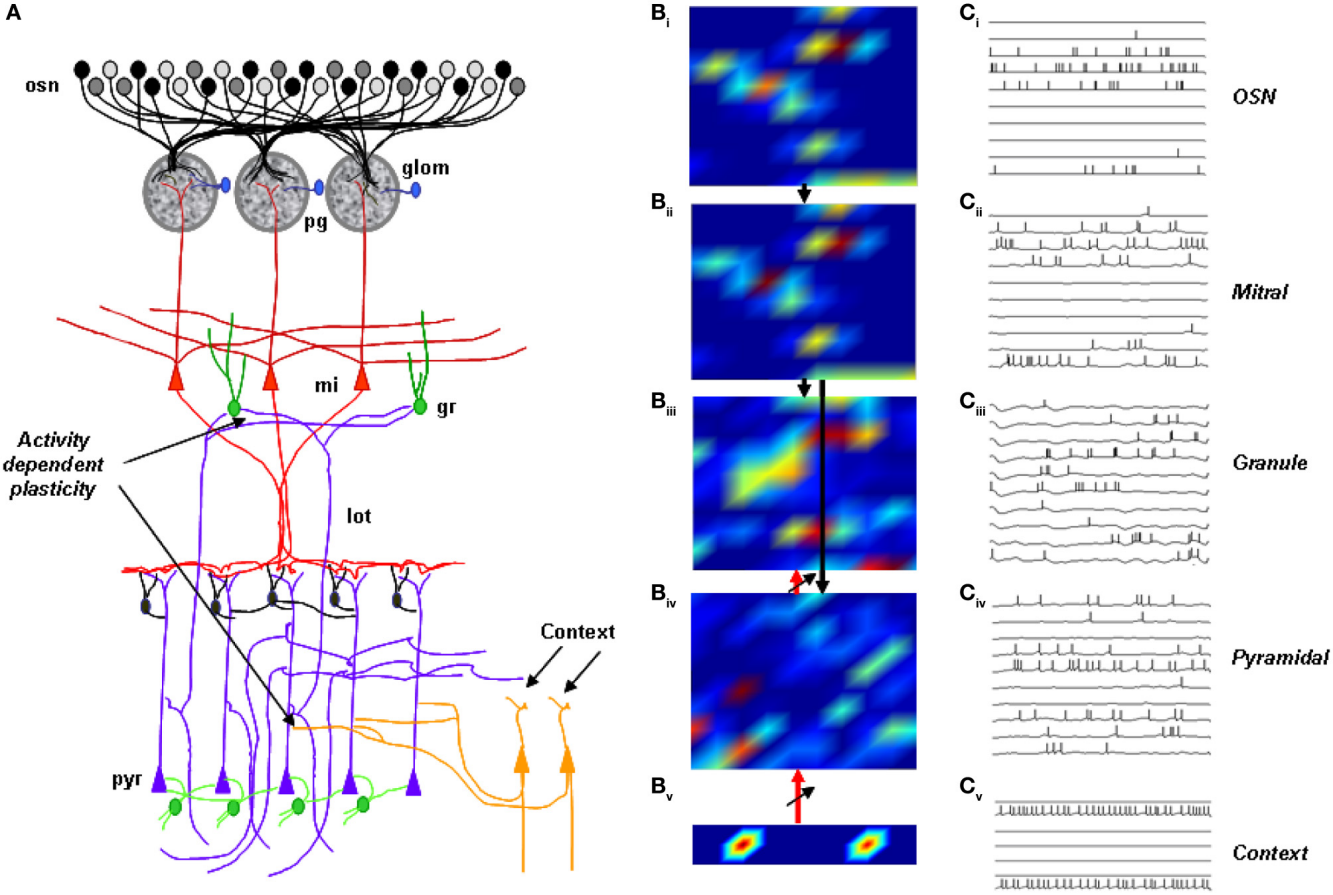
**Computational model of the olfactory bulb. (A)** Sensory neurons (osn) project to glomeruli (glom) and synapses with mitral (mi) and periglomerular cells (pg). Periglomerular cells feed inhibitory synapses to mitral cells. Mitral cell secondary dendrites excite granule cells (gr); granule cells inhibit mitral cells. Mitral outputs project to pyramidal cells; context neurons, responsive to the behavioral context that the mouse is put in, project initially weak synapses exhibiting synaptic plasticity to pyramidal cells. Pyramidal cell (pyr) outputs project back to OB granule cells with initially weak synapses exhibiting plasticity (lot, lateral olfactory tract). **(B)** Neural activity patterns in response to stimulation with a randomly chosen odorant before training. Activity is color-coded from low (blue) to high (red). **(C)** Membrane potential and action potentials of 10 neurons in response to a 200 ms stimulation.

We used a computational model of olfactory sensory neurons, OB and piriform cortex (Figure 2A). The individual elements of this model have been described in detail before (Linster and Cleland, 2001, 2002, 2004; Linster et al., 2003) and have been adapted. Synaptic plasticity between pyramidal cells and granule cells, as described experimentally (Gao and Strowbridge, 2009) is new to this model as is the introduction of “context” neurons (Hasselmo and Wyble, 1997). Context neurons here represent the context of the behavioral experiments, or a combination of features of the cage in which odor exposure happened. These context neurons, after training, can drive activity in olfactory cortex, creating context dependent responses as described experimentally (Calu et al., 2007). To enable context learning, synaptic plasticity was also introduced between context neurons and pyramidal cells.

In a model simulating 100 OSNs, 100 mitral (Mi), granule (Gr), periglomerular (PG), 100 pyramidal (Pyr) cells and 10 context neurons, synapses between mitral and pyramidal cells were created randomly with each mitral cell projecting to any pyramidal cell with an equal probability of P_Mit−Pyr_ = 0.1. Intra-cortical connections and interneurons were omitted from this model. Pyramidal cells projected back to randomly chosen granule cells with an overall connection probability of 0.4 and initially weak synapses. 10 context neurons activated by behavioral context connected to pyramidal cells in an all to all fashion with initially weak synapses. Information flow in the model is both feedforward (OSNs activate mitral cells, mitral cells activate granule cells and cortical pyramidal cells) and feedback (context cells activate pyramidal cells and pyramidal cells activate granule cells). Representative spatial activation patterns and neural firing patterns are depicted in Figures 2B,C.

In the simulations presented here (Figure 2B), simulated exposure to an odorant in a specific context induced activity dependent plasticity of synapses from pyramidal to granule and from context to pyramidal cells. Synaptic strengths were first calculated from the parameters given in Table 1, and responses to simulated odorants were obtained. To simulate perceptual learning in response to repeated exposure to an odorant, synapses between pyramidal and granule and between context and pyramidal cells underwent synaptic potentiation:

$$w_{ij}^{trained}=w_{ij}^{naive}+\eta *\sum_{i,j\;=\;0}^{N}x_{i}x_{j}$$

where *w*_*ij*_ is the synaptic strength between the presynaptic pyramidal cell (context cell) *j* and the postsynaptic granule cell (pyramidal cell) *i*, η is the rate of potentiation and *x*_*j*_ and *x*_*i*_ are the total numbers of spikes emitted by the pre and postsynaptic cells during the period of odor stimulation.

**Table 1:** **Model parameters**.

Olfactory sensory neurons (OSN)	τ = 5.0 ms; τ_min_ = 0.0; τ_max_ = 8.0
Mitral cells (Mi)	τ = 4.0 ms; τ_min_ = −0.01; τ_max_ = 8.0
Granule cells (Gr)	τ = 4.0 ms; τ_min_ = −1.5; τ_max_ = 8.0
Afferent, OSN to Mi	w_OSN-Mi_ = 0.028; E_N,OSN-Mi_ = +70; *t*_1_ = 1.0; *t*_2_ = 2.0
Secondary dendrites, Mi to Gr	w_Mi-Gr_ = 0.003; E_N,Mi-Gr_ = 70; *t*_1_ = 1.0; *t*_2_ = 2.0
Feedback inhibitory, Gr to Mi	w_Gr-Mi_ = 0.01; E_N,Gr-Mi_ = −5; *t*_1_ = 4.0; *t*_2_ = 8.0
Feedback inhibitory, Gr to Gr	w_Gr-Gr_ = 0.1; E_N,Gr-Gr_ = −5; *t*_1_ = 4.0; *t*_2_ = 8.0
Mitral cell to pyramidal cell	*p* = 0.25; w_Mi-Pyr_ = 0.043; E_Mi-Pyr_ = 70; *t*_1_ = 1.0; *t*_2_ = 2.0
Pyramidal cell association fibers	*p* = 0.1; w_Pyr-Pyr_ = 0.0001; E_Pyr-Pyr_ = 70; *t*_1_ = 1.0; *t*_2_ = 2.0
Pyramidal cell to granule cell	*p* = 0.4; w_Pyr-Pyr_ = 0.002; E_Pyr-Pyr_ = 70; *t*_1_ = 1.0; *t*_2_ = 2.0
Context cell to pyramidal cell	*p* = 1.0; w_Pyr-Pyr_ = 0.002; E_Pyr-Pyr_ = 70; *t*_1_ = 1.0; *t*_2_ = 2.0

## Results

### Contextual priming produces similar patterns of responsiveness in the OB as odor stimulation

**Figure 3:**
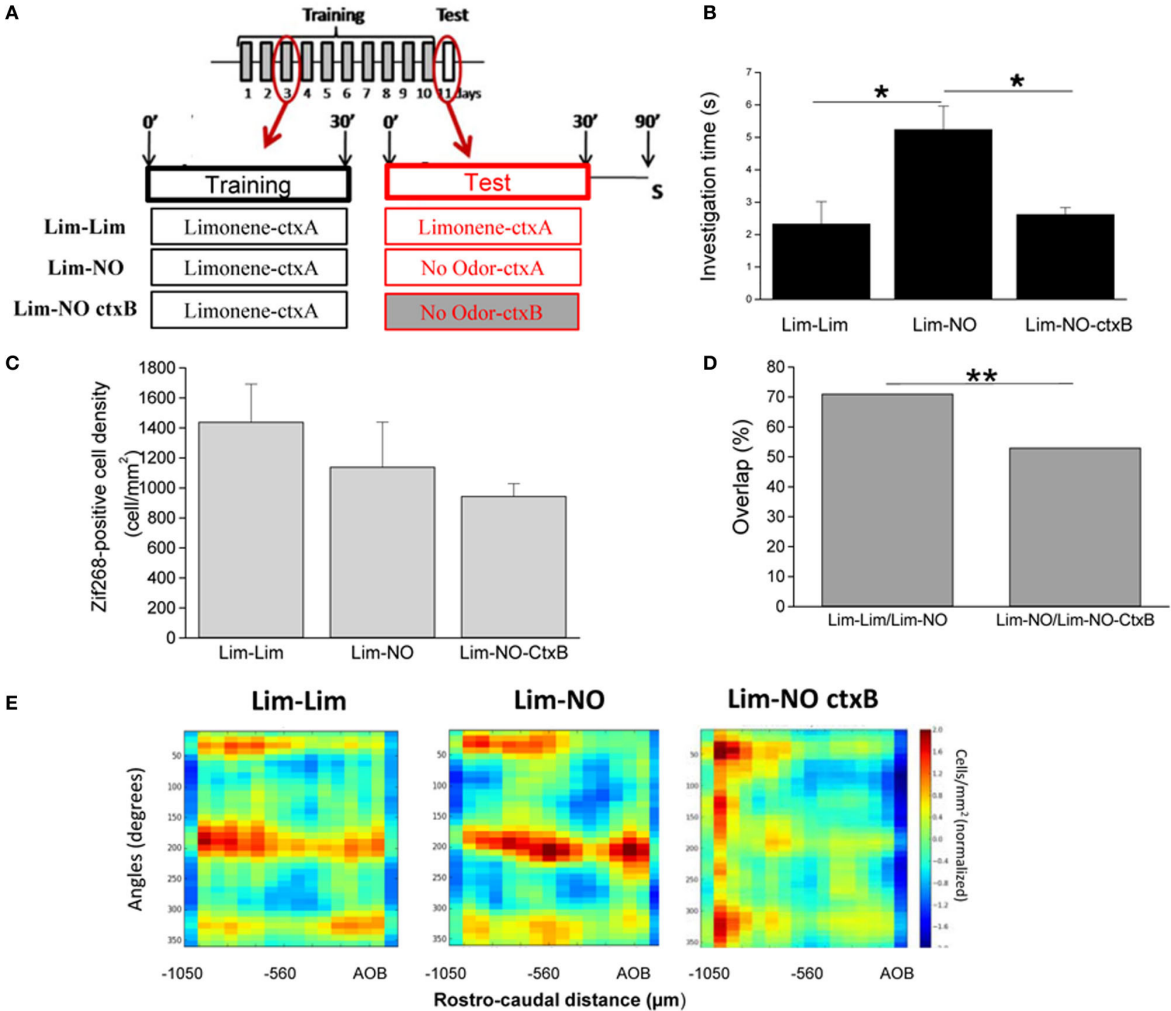
**Context-evoked behavioral and OB neural responses in the absence of olfactory stimulus. (A)** Mice were trained to associate a visual context to an odorant (+limonene: Lim) 30 min per day during 10 days and tested on day 11. The day of the test, the same odorant as during training (“Lim-Lim” group), or an empty swab (“Lim-NO” group, NO: no odor) was introduced in the cage. In a third experimental group, mice were trained with +limonene, but tested in a different context without the odorant (“Lim-NO-ctxB” group). Mice were sacrificed (S) 1 h after the test. **(B)** Investigation time of the odor delivery device. Mice trained during 10 days with Lim and tested with no odor (Lim-NO) showed an investigation time of the odor port superior to the mice trained and tested with the same odorant (Lim-Lim). Moreover, when the visual context changed the day of the test, the increase of investigation time was no longer observed. **(C)** The density of Zif268-positive cells was similar between all groups. **(D)** The overlap between maps of Lim-Lim and Lim-NO was high (72%) indicating the similarity between those two maps. Overlap significantly decreased when Lim-NO was compared to Lim-NO-ctxB. (^*^p < 0.05; ^**^p < 0.005). **(E)** Normalized 2-D maps of the density of Zif268-positive cells in the granule cell layer of Lim-Lim and Lim-NO groups showed a similar pattern of Zif268 expression. Cell density in this figure and in following figures is color-coded from low (blue) to high (red). When the context was changed the day of the test, this pattern was altered (Lim-NO-ctxB).

To test for contextual activation of OB neurons, mice were trained to associate an odorant (+limonene) with a visual context (visual cues added to a transparent cage, context A) by being introduced into the cage for 30 min per day during 10 consecutive days (Figure 3A). On day 11 (test), one group of mice was placed in the same visual context with the same odorant (Lim-Lim, ctxA), a second group of mice was placed in the same visual context with no odorant (Lim-NO, ctxA) and a third group was placed in novel visual context (context B) with no odorant (Lim-NO-ctxB) (Figure 3A). Upon testing, the group exposed to the visual context only (Lim-NO) investigated the odor delivery device significantly more than both other groups (Lim-Lim and Lim-NO-ctxB) (group effect *p* = 0.008; Lim-Lim vs. Lim-NO *p* = 0.011, Lim-NO vs. Lim-NO-ctxB *p* = 0.004) (Figure 3B), suggesting an expectation of the stimulus. This expectation was specific to the context previously associated with the odor stimulus, because the increased sniffing did not occur in the novel context (Lim-No ctx B) (Figure 3B). At the neural level, the overall Zif268-positive cell density in the granule cell layer of the OB did not vary among the three groups [*F*_(2, 12)_ = 1.16, *p* = 0.34] (Figure 3C); however, the similarity between patterns evoked by the training context only (Lim-NO) and odor stimulus (Lim-Lim) was high (72% overlap), whereas overlap between patterns evoked by the training context (Lim-NO) and a new context (Lim-NO-ctxB) was significantly less (47% overlap, *p* < 0.0001) (Figures 3D,E). These findings show that the training context previously associated with the odorant induced a pattern of activity mimicking odor specific activity as well as a significant behavioral response.

### Context-evoked neural activation patterns are specific to the associated odor

**Figure 4:**
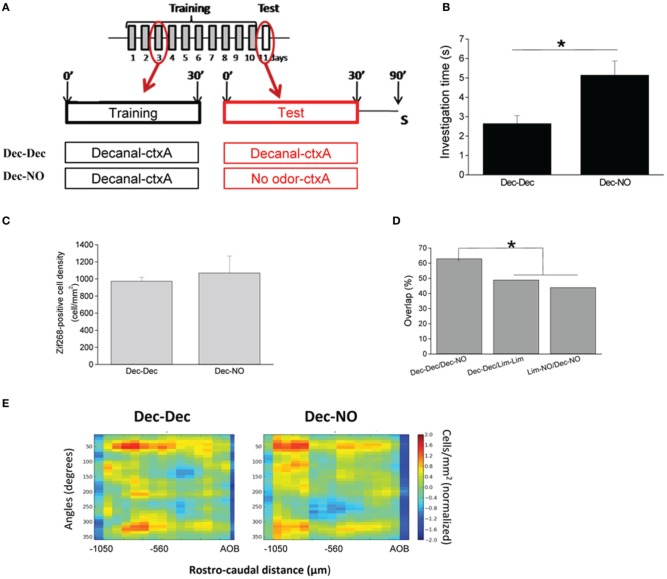
**Context-evoked neural activation patterns in the OB are specific to the associated odor. (A)** Mice were trained to associate a context to an odorant, the decanal (Dec), 30 min per day during 10 days. The day of the test, the same odorant as during training (Dec-Dec), or an empty swab (Dec-NO) was introduced in the cage. Mice were sacrificed (S) 1 h after the test. **(B)** Mice trained during 10 days with Dec and tested with no odor (Dec-NO) showed an investigation time of the odor port superior to the mice trained and tested with the odorant (Dec-Dec). **(C)** The density of Zif268-positive cells was similar between both groups. **(D)** The overlap between maps of Dec-Dec and Dec-NO was high (66%) indicating the similarity between those two maps. Overlaps significantly decreased when other comparisons were performed. **(E)** Normalized 2-D maps of the density of Zif268-positive cells in the granule cell layer of the OB of Dec-Dec and Dec-NO groups showed similar patterns of Zif268 expression. (^*^p < 0.05).

We confirmed this result with an additional set of mice in which context A was associated with a different odorant (decanal, Figure 4A). Results were similar to those found with the previous odorant (Figure 3). Indeed, presentation of the context alone (Dec-NO) induced a significantly increased investigation of the odor delivery device (*p* = 0.01) (Figure 4B), suggesting an expectation of the odorant. As with limonene, overall levels of Zif268 expression were not significantly different between the two groups (Figure 4C). The patterns of neural activation in the OB evoked by the context alone (Dec-NO) were very similar to those evoked by the odor in the same context (Dec-Dec) (62% overlap) (Figures 4D,E). In contrast, the patterns evoked in mice who associated decanal with context A were significantly different from those evoked by context A in mice who associated context A with limonene [44% overlap between Dec-NO and Lim-NO and 43% between Dec-Dec and Lim-Lim compared to the 62% overlap between Dec-Dec and Dec-NO (*p* < 0.0004) or compared to the 72% overlap between Lim-NO and Lim-Lim (*p* < 0.0001)], showing that the activity pattern evoked by context A alone was specific to the odor associated with that context by the mice.

### Context-evoked neural activation in anterior piriform cortex is specific to the associated context

**Figure 5:**
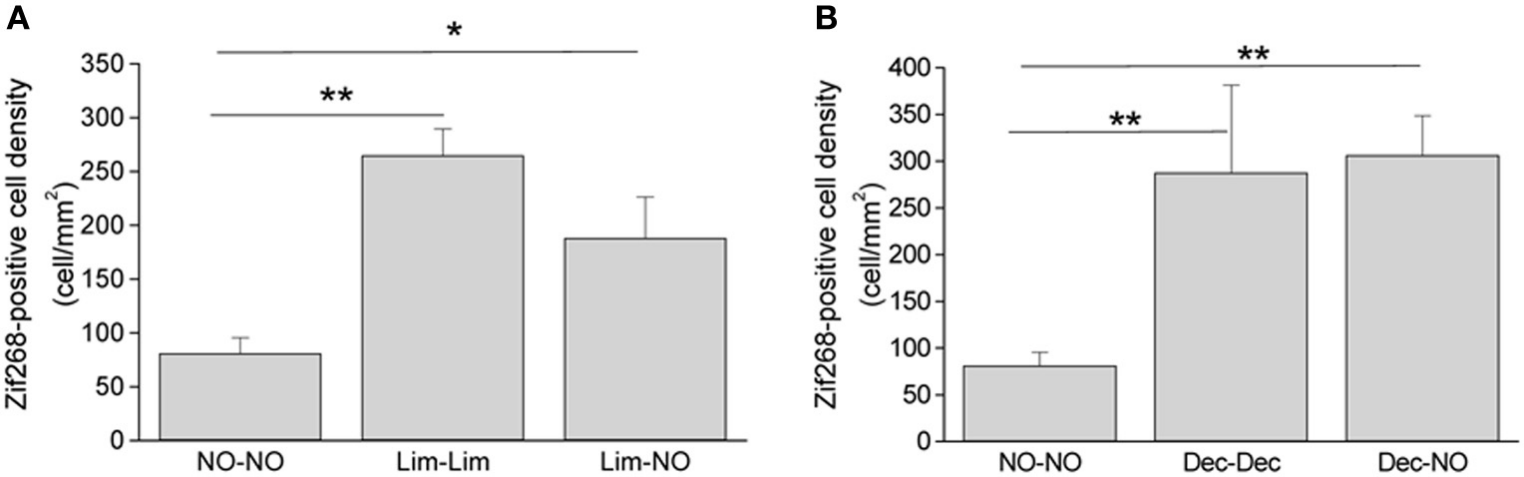
**Context-evoked activity in the piriform cortex. (A)** Zif268-positive cell density in the piriform cortex was increased in response to odorant stimulation (Lim-Lim) and in response to context (Lim-NO) compared to control animals (“NO-NO” group). **(B)** Same as A, with decanal used instead of limonene (^*^p < 0.05, ^**^p < 0.005).

In anterior piriform cortex, to which the OB projects (Shipley and Adamek, 1984; Haberly, 1998), we found that Zif268-positive cell density did not differ between groups smelling the odor and groups exposed to the associated context alone. Because no specific spatial activity pattern is associated with an odorant in cortex (Isaacson, 2010) we did not analyze the overlap between maps but rather compared to a naive control group not exposed to an odorant during training and testing (NO-NO) and found more activation in the limonene-context associated groups compared to naive [*F*_(2, 6)_ = 13.70, *p* = 0.006, NO-NO vs. Lim-Lim *p* = 0.002; NO-NO vs. Lim-NO *p* = 0.019] (Figure 5A). The same results were obtained in the group of mice presented with decanal during context association [*F*_(2, 5)_ = 18.07, *p* = 0.005, NO-NO vs. Dec-Dec *p* = 0.004; NO-NO vs. Dec-NO *p* = 0.004] (Figure 5B).

### Context-activated behavioral and neural responses depend on central inputs to the olfactory bulb

**Figure 6:**
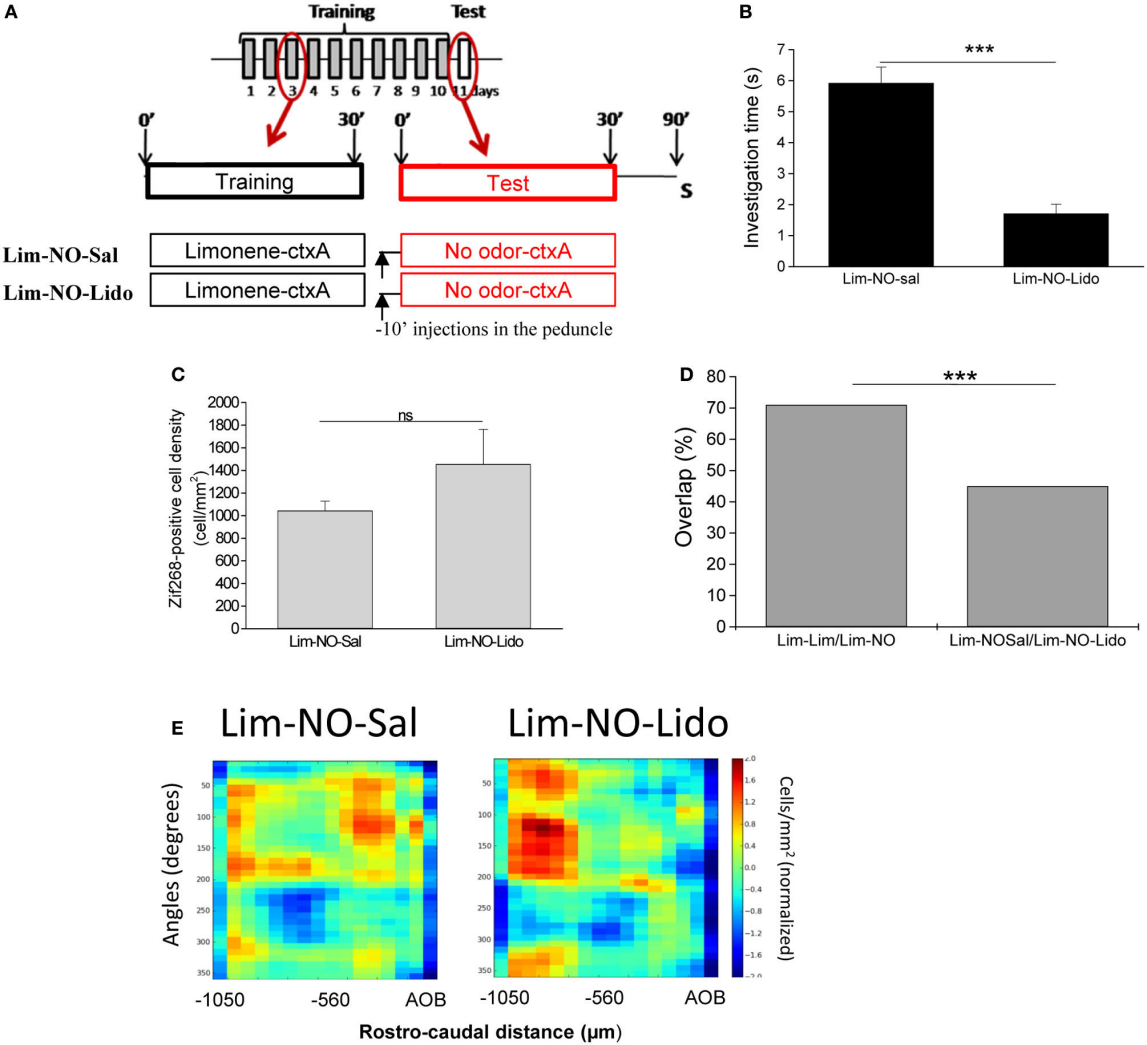
**Context-activated behavioral and neural responses depend on central inputs to the olfactory bulb. (A)** Mice were trained similarly to previous groups (see Figure 1) and tested with no odor. The day of the test, animals were injected in the olfactory peduncle with lidocaine (“Lim-NO-Lido” group) or saline (“Lim-NO-Sal” group) 10 min before being placed in their training cage. **(B)** Lidocaine injection induced a decrease of investigation time compared to animals injected with saline. **(C)** The density of Zif268-positive cells was similar between both groups. **(D)** The overlap between Lim-Lim and Lim-NO significantly decreased when feedbacks to the OB were altered (^***^p < 0.0001). **(E)** The spatial pattern of Zif268-positive cell density was altered when the animals were injected with lidocaine.

Information about a learned context is likely to be transmitted as top-down information to the OB (Gilbert and Sigman, 2007). Mice with surgically implanted cannula in the medial olfactory peduncle were exposed to the visual context and odorant during 1 h daily for 10 days. On day 11, mice were exposed to the context alone. In this experimental group, lidocaine (or saline) was infused into the olfactory peduncle in such a manner as to decrease central inputs to the OB without affecting olfactory input (Martin et al., 2006) (Figure 6A). In response to the context, lidocaine-induced decrease of peduncle activity (Lim-NO-Lido) led to a significant decrease in context-induced investigation time compared to saline-infused animals (Lim-NO-Sal) (*p* < 0.0005) (Figure 6B). The overall level of Zif268 expression in the OB was not significantly different between animals infused with lidocaine or saline (*t*-test, *p* = 0.11) (Figure 6C), suggesting no direct effect of lidocaine in the OB through diffusion from the infusion site. However, the distribution of Zif268-positive cells was deeply altered in Lim-NO-Lido compared to Lim-NO-Sal (47% overlap between Lim-NO-Lido and Lim-NO-Sal vs. 72% overlap between Lim-NO and Lim-Lim, *p* < 0.0001) (Figures 6D,E). This result shows that context evoked activity in the OB depends on functional feedback inputs from other brain centers.

### Computational modeling of the neural circuits underlying context-driven OB activation

**Figure 7:**
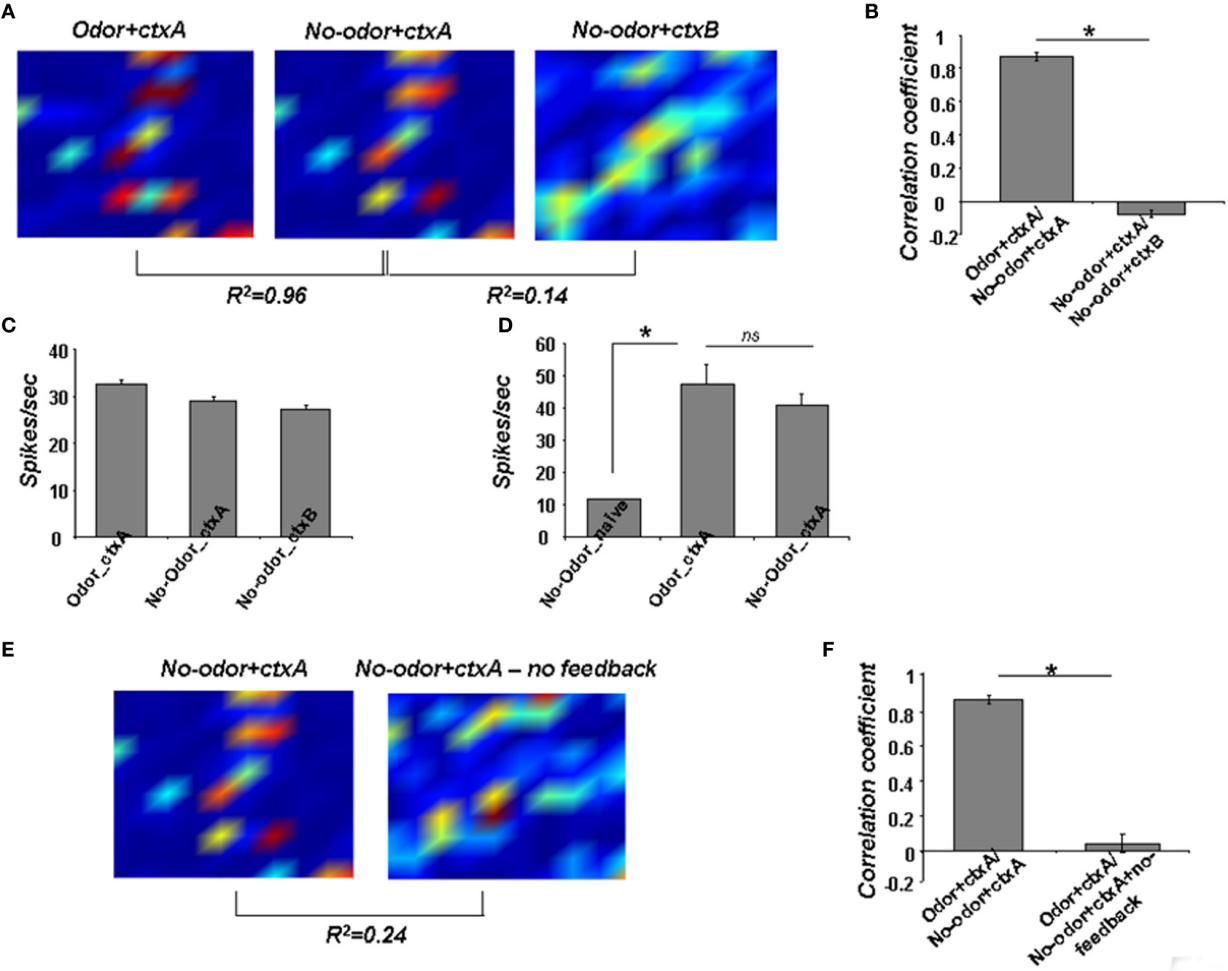
**Computational modeling of the neural circuits underlying context-driven OB activation. (A)** Odor representations in granule cells after training on a randomly chosen odor [mean activity over 200 ms; odor and context (Odor+ctxA), trained context only (No-odor+ctxA), novel, untrained context (No-odor+ctxB)]. **(B)** Correlation between post-training activation patterns (Odor+ctxA/No-odor+ctxtA and No-odor+ctxA/No-odor+ctxB). **(C)** Spikes per seconds evoked in granule cells after training in response to the trained odorant and context (Odor+ctxA), the trained context only (No-odor+ctxA), and a novel context (No-odor+ctxB). **(D)** Spikes per second evoked in pyramidal cells in a naive model exposed to context only (before training; odor naive, No-Odor_naive), to the trained odorant/context (Odor+ctxA), or to the trained context only (No-odor+ctxA). **(E)** Odor representations in granule cells in response to stimulation with the trained context only with the intact (No-odor+ctxA) or lesioned (No-odor+ctxA-no feedback) feedback. **(F)** Correlation between Odor+ctxA/No-odor+ctxtA and Odor+ctxA/No-odor+ctxA+no-feedback. (^*^p < 0.05).

We then used a well-described computational model of the olfactory system (Linster et al., 2007; Linster and Cleland, 2009), to which we added an abstract set of context neurons encoding the visual features of the context (Figures 2A–C) (Gao and Strowbridge, 2009) and a connection between context neurons and cortical pyramidal cells. The behavioral association between the context and odor was simulated by stimulating olfactory sensory neurons with an “odor” while simultaneously stimulating the context neurons with a “context;” during the formation of this association the activity-dependent learning rule is turned on (Figures 2B,C). After training, stimulation with odorant and the context drove granule cell activation (Figure 7A, Odor+ctxA, corresponding to Lim-Lim in experimental data). Presence of the trained context only (Figure 7A; No-odor+ctxA, corresponding to Lim-NO in experimental data) stimulated a very similar pattern of activity (*r*^2^ = 0.96) (Figure 7A). When a novel context was presented to the network in the absence of odorant, pyramidal cells were not activated and did not shape granule cell activation: the activation pattern of granule cells was non-specific and the overlap with the representation triggered by the trained context was low (*r*^2^ = 0.14, Figure 7A, No-odor-ctxb corresponding to NO-Lim-ctx B in experimental data). A set of ten simulations with novel networks and randomly chosen odorants confirmed these results to be independent of the choice of odorant. There was a statistically significant difference between the two sets of overlap [*F*_(1, 17)_ = 816.16; *p* < 0.001], showing that while the trained context evokes granule cell activity resembling that in response to the odorant, the novel context does not (Figure 7B). We observed a significant effect of group on the discharge rates of granule cells (no-odor naive, odor+ctxA, no-odor+ctxA; [*F*_(2, 27)_ = 21.285, *p* < 0.001] (Figure 7C); with individual significant differences between the naive network and both trained networks (*p* < 0.001) but not between the trained networks (*p* > 0.2) (Figure 7C), as observed experimentally (Figure 3). Cortical pyramidal cells exhibited significantly higher spike rates in response to the trained odor (Figure 7D, Odor_ctxA) or the context alone (Figure 7D, N-Odor_ctx) than in response to no odor in an untrained network (Figure 7D, No-Odor_naive), as shown experimentally (Figure 5). When cortical feedback inactivation were simulated by decreasing the synaptic weights from pyramidal neurons to granule cells, the overlap between granule cell activation patterns in response to context alone with intact feedback (No−odor+ctxA) and context alone with “lesioned” feedback (No-odor+ctxA-no feedback) was very low (*r*^2^ = 0.24 in the example in Figures 7E,F).

## Discussion

In this study, in order to trigger an internal odor representation, we developed a behavioral paradigm which allowed inducing odor expectations by exposing animals to a context previously associated to an odorant. The increase in investigation time directed to the empty odor source the day of testing strongly supports the view that animals actually expected, and even searched for the odorant.

Using this original paradigm, we demonstrated a patterned, odor and context specific activation of olfactory cortices by contextual information in the absence of a physical stimulus. This result is in accordance with a previous fMRI study in humans, in which the same brain regions were activated by imagining visual (Halpern and Zatorre, 1999), auditory (Kosslyn et al., 2001), or olfactory stimuli (Bensafi et al., 2007) and actually viewing, hearing or smelling them. In humans, the previous studies showed that imagining odors activated olfactory structures as the piriform cortex, left insula and amygdala. Here, we showed that not only the piriform cortex but also the OB were activated during context evoked odor expectation. This finding is reminiscent of the “search image” revealed by EEG recordings in rabbits (Freeman, 1983). The “search image” was defined by Freeman as a large-scale pattern of strengthened connections (synaptic template) that could serve to represent an expected stimulus even if it is not present. We mapped Zif268 expression in granule cells because bulbar patterned expressions are odor-specific and replicable across individuals in these cells (Inaki et al., 2002; Mandairon et al., 2006; Busto et al., 2009) and hence allowed us to compare odor and context evoked activation patterns. We found that the context induced activation pattern in the OB was highly similar to the one observed after odor stimulation and was specific to each odorant tested. Taken together, these findings strongly suggested that odor expectation induced an internal neural representation of the odorant in the OB which was context and odor specific and resulted from the specific odor-context association the animal was exposed to.

Both behavioral and OB neural context-driven responses depend on intact centrifugal projections to the OB. When we blocked top-down fibers by infusing mice with an anesthetic (Lidocaine) in the medial part of the olfactory peduncle 10 min before testing we eliminated the context driven behavioral and neural responses. The olfactory peduncle contains fibers projecting from the rest of the brain to the OB including the anterior olfactory nucleus, glutamatergic fibers from the piriform and entorhinal cortices and cortical amygdaloid nuclei (Haberly and Price, 1977, 1978), cholinergic neurons from basal forebrain or noradrenergic (McLean and Shipley, 1991), serotoninergic (McLean and Shipley, 1987) neurons as well as sparse projections from the hypothalamus. We assume that a high percentage of these incoming fibers are blocked by our lidocaine injection; we can therefore not speculate on which centrifugal fibers contribute to our observations. From a computational point of view, any secondary or tertiary olfactory structures receiving odor and context information and projecting back the OB could perform this function. Overall, we observed that top-down inactivation resulted in a decreased investigation time compared to non-injected mice, as if they were not expecting the odor. This result is consistent with data showing that blockade of central inputs to the OB using an infusion of Lidocaine in the olfactory peduncle reduced the amplitude of odor-induced oscillatory beta responses (Martin et al., 2006) which are involved in odor associative learning and in anticipation of odor stimuli (Kay et al., 2009). The simulations presented showed that a combined model of OB, olfactory cortex and context neurons, including experimentally described synaptic plasticity between pyramidal and granule cells (Gao and Strowbridge, 2009) can reproduce the described priming of granule cell activation by visual context. The model suggests that during the learning of the odor-context association, information flows from bulb to cortex and from cortex to bulb, and that activity dependent plasticity in both pathways can suffice to support context-driven bulbar activity in the absence of olfactory stimulation. The association with context needs to be performed outside the OB to prevent changes in odor processing in the absence of the learned context. Piriform cortex is only one of many candidate structures to perform this function; it was chosen for these simulations because (a) cortical pyramidal cells project onto bulbar granule cells with synapses that have been shown to undergo activity dependent plasticity (Gao and Strowbridge, 2009) and (b) because neural activity in piriform cortex has been shown to be modulated by behavioral context (Calu et al., 2007). In theory, any brain area receiving odor inputs from the OB and projecting back to OB granule cells and capable of forming an association with context information would yield equivalent results. Our simulations results are not dependent in a specific structure being implemented.

The data presented, together with the computational results suggest that both odor and contextual information shape rather than create OB neural responses. Odor quality, or the expectation thereof, would therefore be encoded in the spatio-temporal patterns of bulbar activity (Kay et al., 2009; Mori and Sakano, 2011) and can be elicited by sensory or central inputs.

### Conflict of interest statement

The authors declare that the research was conducted in the absence of any commercial or financial relationships that could be construed as a potential conflict of interest.
